# Soft tissue masses: distribution of entities and rate of malignancy in small lesions

**DOI:** 10.1186/s12885-020-07769-2

**Published:** 2021-01-22

**Authors:** Felix G. Gassert, Florian T. Gassert, Katja Specht, Carolin Knebel, Ulrich Lenze, Marcus R. Makowski, Rüdiger von Eisenhart-Rothe, Alexandra S. Gersing, Klaus Woertler

**Affiliations:** 1 Department of Diagnostic and Interventional Radiology, School of Medicine & Klinikum rechts der Isar, Technical University of Munich, Ismaninger Str. 22, 81675 Munich, Germany; 2 Department of Pathology, School of Medicine & Klinikum rechts der Isar, Technical University of Munich, Munich, Germany; 3 Department of Orthopaedic Surgery, School of Medicine & Klinikum rechts der Isar, Technical University of Munich, Munich, Germany

## Abstract

**Background:**

Small soft tissue masses are often falsely assumed to be benign and resected with failure to achieve tumor-free margins. Therefore, this study retrospectively investigated the distribution of histopathologic diagnosis to be encountered in small soft tissue tumors (≤ 5 cm) in a large series of a tertiary referral center.

**Methods:**

Patients with a soft tissue mass (STM) with a maximum diameter of 5 cm presenting at our institution over a period of 10 years, who had undergone preoperative Magnetic resonance imaging and consequent biopsy or/and surgical resection, were included in this study. A final histopathological diagnosis was available in all cases. The maximum tumor diameter was determined on MR images by one radiologist. Moreover, tumor localization (head/neck, trunk, upper extremity, lower extremity, hand, foot) and depth (superficial / deep to fascia) were assessed.

**Results:**

In total, histopathologic results and MR images of 1753 patients were reviewed. Eight hundred seventy patients (49.63%) showed a STM ≤ 5 cm and were therefore included in this study (46.79 +/− 18.08 years, 464 women). Mean maximum diameter of the assessed STMs was 2.88 cm. Of 870 analyzed lesions ≤ 5 cm, 170 (19.54%) were classified as superficial and 700 (80.46%) as deep. The malignancy rate of all lesions ≤ 5 cm was at 22.41% (superficial: 23.53% / deep: 22.14%). The malignancy rate dropped to 16.49% (20.79% / 15.32%) when assessing lesions ≤ 3 cm (*p* = 0.007) and to 15.0% (18.18% / 13.79%) when assessing lesions ≤ 2 cm (*p* = 0.006).

Overall, lipoma was the most common benign lesion of superficial STMs (29.41%) and tenosynovial giant cell tumor was the most common benign lesion of deep STMs (23.29%). Undifferentiated pleomorphic sarcoma was the most common malignant diagnosis among both, superficial (5.29%) and deep (3.57%) STMs.

**Conclusions:**

The rate of malignancy decreased significantly with tumor size in both, superficial and deep STMs. The distribution of entities was different between superficial and deep STMs, yet there was no significant difference found in the malignancy rate.

## Background

Malignant soft tissue tumors account for approximately 1% of all adult and approximately 20% of all pediatric solid malignant cancers [1, 2]. The most common entities among malignant soft tissue sarcomas are liposarcoma, leiomyosarcoma, and undifferentiated pleomorphic sarcoma [3]. In the 5th edition of the classification of soft tissue tumors, the World Health Organisation (WHO) defined several categories of soft tissue lesions with further subcategories and indicated their dignity with either benign, intermediate (locally aggressive or rarely metastasizing) or malignant [4].

The T1 stage of malignant tumors is defined by the American Joint Committee on Cancer (AJCC) as a lesion with a diameter < 5 cm [5]. Although malignant lesions are usually larger in size, small soft tissue masses (STMs) still comprise a considerable proportion of malignant soft tissue tumors [6]. Datir et al. showed that approximately 10% of malignant STMs were staged as T1 tumors at initial diagnosis, which correlates with a maximum tumor size of 5 cm [7].

In clinical practice, smaller lesions are often assumed to be benign and therefore, commonly undergo inadequate resection outside specialized tumor centers. Furthermore, current guidelines include resection-biopsy for soft tissue lesions with a diameter of less than 3 cm, possibly leading to an excision of malignant tumors with failure to achieve tumor-free margins [8, 9]. It is therefore highly relevant to know the precise percentage of malignancies in small soft tissue lesions and to be able to estimate the distribution of expected entities even without having performed advanced imaging.

A study published by Pham et al. showed a malignancy rate of 21% of soft tissue tumors smaller than 2 cm, excluding simple lipomas, juxta-articular cysts, metastases, and soft tissue

lesions without histological confirmation [10]. Nevertheless, the sample size of their series was small (*n* = 42), and no further differentiation according to tumor sizes was performed. To our knowledge, there was no recent study performed that evaluated the distribution of STMs in a larger study cohort.

To improve differentiation between benign and malignant STMs on Magnetic resonance (MR) imaging, Obaid et al. created a risk stratification model including criteria, such as edema, intralesional fat and signal intensity patterns as well as lesion size in a series of 110 patients [11]. Although average malignant lesions showed a larger diameter, approximately 15% of lesions with a diameter of less than 5 cm were malignant in their study. Consequently, especially in small lesions a more precise evaluation of the distribution of tumor entity and rate of malignancy, depending on the maximum diameter, could help to determine further diagnostic workup and treatment.

Therefore, in this study we aimed to determine the rate of malignancy depending on tumor size and assessed the distribution of the entities of soft tissue tumors with a maximum diameter of less than 5 cm, 3 cm and 2 cm.

## Methods

### Subjects

Approval of the Institutional Review Board was obtained prior to this study. Written informed consent was waived for this retrospective analysis of routinely acquired imaging and clinical data. Over a period of 10 years (January 2010 through March 2020) all patients with a STM presenting at our tertiary care center, who received biopsy or / and surgical resection with a final histopathological diagnosis, were selected from our musculoskeletal database for further analysis (*n* = 2172).

### Image acquisition and diameter evaluation

Imaging of the soft tissue mass was performed on either a 1.5 or a 3 Tesla MR scanner in all subjects prior to surgery using various protocols. All MR protocols included sequences in both, the short and the long axis allowing for proper evaluation of tumor diameter and location with respect to the deep fascia. Patients without available preoperative MR images were excluded from this study (*n* = 374). In the remaining 1978 patients, the precise maximum tumor diameter was determined on MR images by one radiologist (F.G.G.). All available cases with a maximum diameter of 5 cm and smaller were included in this study (*n* = 870). Besides histopathological diagnosis of the STMs, tumor location was extracted from the database including the categories “Head/Neck”, “Trunk”, “Upper Extremity” (without hand), “Lower Extremity” (without foot), “Hand” and “Foot”. Additionally, analysis of tumor depth in relation to the fascia (superficial / deep to fascia) and differentiation between patients younger than 18 years (age group A), patients between 18 and 40 years (age group B) and patients older than 40 years (age group C) was performed and included in this study. For evaluation of intra-reader reproducibility, the tumor diameter of 20 lesions was reevaluated after 14 days.

### Assessment of STMs

A final histopathological diagnosis was available in all of these selected cases. Classification of lesions was adjusted to the 5th edition of classification of tumors of soft tissue and bone of the WHO according to the final histologic diagnosis [4]. Lesions in the intermediate category capable of local recurrence but not capable of distant metastasis were considered as benign for this analysis. Low-grade liposarcomas/atypical lipomatous tumors were excepted from this proceeding and summarized under the diagnosis “liposarcoma”. A separate analysis of low- and high-grade liposarcomas was however performed as well.

Some lesions represented in our database were identified as sarcomas, but further histological specification was not possible / performed at the time of diagnosis. These lesions were assigned to the category “Sarcoma not otherwise specified”. Differentiation between malignant and benign lesions was also performed according to the WHO classification. In total, 21 malignant and 16 benign histopathological entities were used for this analysis. Additionally, further histopathological specification of non-neoplastic masses was performed.

### Statistical analyses

All statistical analyses were performed using R version 3.2.4 (R Foundation for Statistical Computing). Method of least squares was applied for Regression analyses of malignancy rate as a function of diameter. Chi-square test was performed in order to analyse the differences in malignancy rates and Wilcoxon rank-sum in order to analyse the differences in patient age between the ≤ 5 cm and the ≤ 3 cm / ≤ 2 cm group. For all analysis, a *p*-value of ≤ 0.05 was considered significant. For the evaluation of the intra-reader reproducibility, determination of the maximum diameter was repeated after 14 days and Cohen’s Kappa was calculated.

## Results

### Demographics

Of the 870 patients with a lesion ≤5 cm included in our study, 406 (46.67%) were male and 464 (53.33%) were female with a mean age of 46.79 +/− 18.08 years. 46 (5.29%) of the patients were under the age of 18 years, 289 (33.22%) between 18 and 40 years and 535 (61.49%) older than 40 years. Among the 473 patients with lesions ≤3 cm, 277 (58.56%) were female and the mean age was 46.04 +/− 16.9 years, and among the 240 patients with lesions ≤ 2 cm, 143 (59.58%) were female and the mean age was 44.87 +/− 16.72 years. No significant differences were observed comparing distribution of gender for STMs ≤5 cm vs. STMs ≤3 cm (*P* = 0.08) and for STMs ≤5 cm vs. STMs ≤2 cm (*P* = 0.09) as well as age (*P* = 0.56 and *P* = 0.16, respectively) between the three groups.

### Distribution of tumor entities depending on size, depth, location and patient age

The mean maximum tumor diameter across all STMs ≤5 cm was 2.88 cm (STMs ≤3 cm: 1.99 cm; STMs ≤2 cm: 1.46 cm). Reevaluation of maximum tumor diameter of 20 lesions after 14 days showed an excellent intra-reader reproducibility (κ = 0.883). Distribution of tumor entities was analyzed for the STMs ≤5 cm, ≤3 cm and ≤2 cm and sorted into benign, malignant and non-neoplastic masses. Results are shown in Table 1. Tenosynovial giant cell tumor was the most common diagnosis among the benign STMs (19.43% of all STMs ≤5 cm) whereas undifferentiated pleomorphic sarcoma was most common among the malignant STMs (3.91% of all STMs ≤5 cm), independent of the measured maximum tumor diameter. Among the non-neoplastic soft tissue masses, ganglion was the most common diagnosis (7.47% of all STMs ≤5 cm).

**Table 1:** Distribution of tumor entities for all benign, malignant and non-neoplastic soft tissue masses ≤5 cm, ≤3 cm and ≤2 cm included in this study

Benign				Malignant				Non-neoplastic			
Entity	≤5 cm	≤3 cm	≤2 cm	Entity	≤5 cm	≤3 cm	≤2 cm	Entity	≤5 cm	< 3 cm	≤2 cm
Overall	562 (64.6%)	330 (69.77%)	159 (66.25%)		195 (22.41%)	78 (16.49%)	36 (15.0%)		113 (12.99%)	65 (13.74%)	45 (18.75%)
Deep benign fibrous histiocytoma	1 (0.11%)	–	–	Alveolar soft part sarcoma	4 (0.46%)	–	–	Bursitis	6 (0.69%)	4 (0.85%)	3 (1.26%)
Fibroma	33 (3.79%)	26 (5.5%)	16 (6.67%)	Angiosarcoma of soft tissue	6 (0.69%)	2 (0.43%)	–	Ganglion	65 (7.47%)	42 (8.88%)	27 (11.3%)
Fibromatosis	40 (4.6%)	25 (5.5%)	14 (5.83%)	Chondrosarcoma	2 (0.23%)	1 (0.21%)	–	Haematoma	5 (0.57%)	1 (0.21%)	1 (0.42%)
Glomus tumors	1 (0.11%)	–	–	Clear cell sarcoma of soft tissue	3 (0.34%)	1 (0.21%)	1 (0.42%)	Lipoma arborescens	2 (0.23%)	–	–
Haemangioma	74 (8.51%)	48 (10.15%)	20 (8.33%)	Dermatofibrosarcoma protuberans	7 (0.8%)	5 (1.06%)	3 (1.25%)	Pseudotumor	12 (1.38%)	5 (1.06%)	3 (1.26%)
Hamartoma	1 (0.11%)	1 (0.21%)	1 (0.42%)	Epithelioid sarcoma	4 (0.46%)	3 (0.63%)	1 (0.42%)	Others	23 (2.64%)	13 (2.75%)	11 (4.6%)
Hibernoma	1 (0.11%)	–	.	Ewing-Sarcoma	2 (0.23%)	1 (0.21%)	1 (0.42%)				
Lipoma	97 (11.15%)	34 (7.19%)	12 (5.0%)	Extraskeletal osteosarcoma	1 (0.11%)	–	–				
Lymphangioma	1 (0.11%)	–	–	Extrapleural solitary fibrous tumour	6 (0.69%)	1 (0.21%)	1 (0.42%)				
Myopericytoma	3 (0.34%)	2 (0.42%)	2 (0.83%)	Fibrosarcoma	5 (0.57%)	5 (1.06%)	2 (0.83%)				
Myositis ossificans	2 (0.23%)	2 (0.42%)	–	Haemangioendothelioma	1 (0.11%)	1 (0.21%)	1 (0.42%)				
Myxoma	21 (2.41%)	7 (1.48%)	3 (1.25%)	Leyomyosarcoma	18 (2.07%)	10 (2.11%)	6 (2.5%)				
Nodular fasciitis	25 (2.87%)	19 (4.02%)	13 (5.42%)	Liposarcoma ^a^	27 (3.1%)	6 (1.27%)	3 (1.25%)				
Perineurioma	1 (0.11%)	–	–	Lymphoma	7 (0.8%)	1 (0.21%)	–				
Schwannoma	92 (10.57%)	63 (13.32%)	27 (11.25%)	Metastasis	6 (0.69%)	2 (0.42%)	1 (0.42%)				
Tenosynovial giant cell tumor	169 (19.43%)	102 (21.56%)	51 (21.25%)	Malignant peripheral nerve sheath tumor	1 (0.11%)	–	–				
				Myofibroblastic tumor	6 (0.69%)	5 (1.06%)	1 (0.42%)				
				Myxofibrosarcoma	24 (2.76%)	7 (1.48%)	5 (2.08%)				
				Rhabdomyosarcoma	4 (0.46%)	1 (0.21%)	–				
				Synovial sarcoma	20 (2.3%)	9 (1.9%)	3 (1.25%)				
				Undifferentiated pleomorphic sarcoma	34 (3.91%)	12 (2.54%)	5 (2.08%)				
				Sarcoma, not otherwise specified	7 (0.8%)	5 (1.06%)	2 (0.83%)				

^a^of those classified as low-grade liposarcoma/atypical lipomatous tumor: 13 ≤ 5 cm (1.49%), 2 ≤ 3 cm (0.42%), 2 ≤ 2 cm (0.83%)

Regarding the localization of the analyzed STMs, 170 lesions (19.54%) were classified as superficial and 700 as deep (80.46%). The distributions of entities among superficial and deep lesions are shown in Tables 2 and 3. Overall, lipoma was the most common benign lesion of the superficially located STMs (29.41%) and tenosynovial giant cell tumor was the most common benign lesion of the deeply located STMs (23.39%). Undifferentiated pleomorphic sarcoma was the most common malignant diagnosis among both, superficial (5.29%) and deep STMs (3.57%).

**Table 2:** Distribution of tumor entities for benign, malignant and non-neoplastic soft tissue masses ≤5 cm, ≤3 cm and ≤2 cm located superficial to the fascia

Benign				Malignant				Non-neoplastic			
Entity	≤5 cm	≤3 cm	≤2 cm	Entity	≤5 cm	≤3 cm	≤2 cm	Entity	≤5 cm	< 3 cm	≤2 cm
Overall	97 (57.06%)	57 (56.43%)	35 (53.03%)		40 (23.53%)	21 (20.79%)	12 (18.18%)		33 (19.41%)	23 (22.77%)	19 (28.79%)
Deep benign fibrous histiocytoma	1 (0.59%)	–	–	Angiosarcoma of soft tissue	1 (0.59%)	–	–	Ganglion	20 (11.76%)	16 (15.84%)	12 (18.18%)
Fibroma	5 (2.94%)	3 (2.97%)	3 (4.55%)	Dermatofibrosarcoma protuberans	6 (3.53%)	4 (3.96%)	3 (4.55%)	Haematoma	2 (1.18%)	1 (0.99%)	1 (1.52%)
Fibromatosis	11 (6.47%)	9 (8.91%)	7 (10.61%)	Ewing-Sarcoma	1 (0.59%)	1 (0.99%)	1 (1.52%)	Pseudotumor	5 (2.94%)	1 (0.99%)	1 (1.52%)
Haemangioma	11 (6.47%)	9 (8.91%)	6 (9.09%)	Extrapleural solitary fibrous tumor	1 (0.59%)	1 (0.99%)	1 (1.52%)	Others	6 (3.53%)	5 (4.95%)	5 (7.58%)
Hamartoma	1 (0.59%)	1 (0.99%)	1 (1.52%)	Fibrosarcoma	2 (1.18%)	2 (1.98%)	1 (1.52%)				
Hibernoma	1 (0.59%)	–	–	Haemangioendothelioma	1 (0.59%)	1 (0.99%)	1 (1.52%)				
Lipoma	50 (29.41%)	22 (21.78%)	10 (15.15%)	Leyomyosarcoma	4 (2.35%)	1 (0.99%)	1 (1.52%)				
Myxoma	1 (0.59%)	–	–	Liposarcoma ^a^	4 (2.35%)	2 (1.98%)	–				
Nodular fasciitis	1 (0.59%)	1 (0.99%)	1 (1.52%)	Metastasis	3 (1.76%)	1 (0.99%)	1 (1.52%)				
Schwannoma	9 (5.29%)	9 (8.91%)	5 (7.58%)	Myofibroblastic tumor	1 (0.59%)	1 (0.99%)	–				
Tenosynovial giant cell tumor	6 (3.53%)	3 (2.97%)	2 (3.03%)	Myxofibrosarcoma	5 (2.94%)	1 (0.99%)	1 (1.52%)				
				Undifferentiated pleomorphic sarcoma	9 (5.29%)	4 (3.96%)	1 (1.52%)				
				Sarcoma, not otherwise specified	2 (1.18%)	2 (1.98%)	1 (1.52%)				

^a^of those classified as low-grade liposarcoma/atypical lipomatous tumor: 1 ≤ 5 cm (0.59%), 0 ≤ 3 cm, 0 ≤ 2 cm

**Table 3:** Distribution of tumor entities for benign, malignant and non-neoplastic soft tissue masses ≤5 cm, ≤3 cm and ≤2 cm located deep to the fascia

Benign				Malignant				Non-neoplastic			
Entity	≤5 cm	≤3 cm	≤2 cm	Entity	≤5 cm	≤3 cm	≤2 cm	Entity	≤5 cm	< 3 cm	≤2 cm
Overall	465 (66.43%)	273 (73.39%)	124 (71.26%)		155 (22.14%)	57 (15.32%)	24 (13.79%)		80 (11.43%)	42 (11.29%)	26 (14.94%)
Fibroma	28 (4.0%)	23 (6.18%)	13 (7.47%)	Alveolar soft part sarcoma	4 (0.57%)	–	–	Bursitis	6 (0.86%)	4 (1.08%)	3 (1.72%)
Fibromatosis	29 (4.14%)	17 (4.57%)	7 (4.02%)	Angiosarcoma of soft tissue	5 (0.71%)	2 (0.54%)	–	Ganglion	45 (6.43%)	26 (6.99%)	15 (8.62%)
Glomus tumors	1 (0.14%)	–	–	Chondrosarcoma	2 (0.29%)	1 (0.27%)	–	Haematoma	3 (0.43%)	–	–
Haemangioma	63 (9.0%)	39 (10.48%)	14 (8.05%)	Clear cell sarcoma of soft tissue	3 (0.43%)	1 (0.27%)	1 (0.57%)	Lipoma arborescens	2 (0.29%)	–	–
Lipoma	47 (6.71%)	12 (3.23%)	2 (1.15%)	Dermatofibrosarcoma protuberans	1 (0.14%)	1 (0.27%)	–	Pseudotumor	7 (1.0%)	4 (1.08%)	2 (1.15%)
Lymphangioma	1 (0.14%)	–	–	Epithelioid sarcoma	4 (0.57%)	3 (0.81%)	1 (0.57%)	Others	17 (2.43%)	8 (2.15%)	6 (3.45%)
Myopericytoma	3 (0.43%)	2 (0.54%)	2 (1.15%)	Ewing-Sarcoma	1 (0.14%)	–	–				
Myositis ossificans	2 (0.29%)	2 (0.54%)	–	Extraskeletal osteosarcoma	1 (0.14%)	–	–				
Myxoma	20 (2.86%)	7 (1.88%)	3 (1.72%)	Extrapleural solitary fibrous tumour	5 (0.71%)	–	–				
Nodular fasciitis	24 (3.43%)	18 (4.84%)	12 (6.9%)	Fibrosarcoma	3 (0.43%)	3 (0.81%)	1 (0.57%)				
Perineurioma	1 (0.14%)	–	–	Leyomyosarcoma	14 (2.0%)	9 (2.42%)	5 (2.87%)				
Schwannoma	83 (11.86%)	54 (14.52%)	22 (12.64%)	Liposarcoma ^a^	23 (3.29%)	4 (1.08%)	3 (1.72%)				
Tenosynovial giant cell tumor	163 (23.29%)	99 (26.61%)	49 (28.16%)	Lymphoma	7 (1.0%)	1 (0.27%)	–				
				Metastasis	3 (0.43%)	1 (0.27%)	–				
				Malignant peripheral nerve sheath tumor	1 (0.14%)	–	–				
				Myofibroblastic tumor	5 (0.71%)	4 (1.08%)	1 (0.57%)				
				Myxofibrosarcoma	19 (2.71%)	6 (1.61%)	4 (2.3%)				
				Rhabdomyosarcoma	4 (0.57%)	1 (0.27%)	–				
				Synovial sarcoma	20 (2.86%)	9 (2.42%)	3 (1.72%)				
				Undifferentiated pleomorphic sarcoma	25 (3.57%)	8 (2.15%)	4 (2.3%)				
				Sarcoma, not other specified	5 (4.29%)	3 (0.81%)	1 (0.57%)				

^a^of those classified as low-grade liposarcoma/aypical lipomatous tumor: 12 ≤ 5 cm (1.71%), 2 ≤ 3 cm (0.54%), 2 ≤ 2 cm (1.15%)

Furthermore, of the 870 STMs ≤ 5 cm, 35 (4.02%) were located at the head and neck region, 70 (8.05%) at the trunk, 186 (21.38%) at the upper and 401 (46.09%) at the lower extremity, 57 (6.55%) at the hand, and 121 (13.91%) at the foot.

Synovial sarcoma (8.69%) and alveolar soft part sarcoma (6.52%) were the most common tumors in age group A, synovial sarcoma (2.42%), myxofibrosarcoma and dermatofibrosarcoma (both 2.08%) in age group B, and undifferentiated pleomorphic sarcoma (6%) and liposarcoma (4.67%) in age group C.

The most common benign tumors in age groups A and B were tenosynovial giant cell tumor (19.56% / 28.37%) and hemangioma (15.21% / 13.84%), whereas tenosynovial giant cell tumor (14.58%) and lipoma (13.46%) were the most common benign lesions in age group C.

### Malignancy rates

**Fig. 1:**
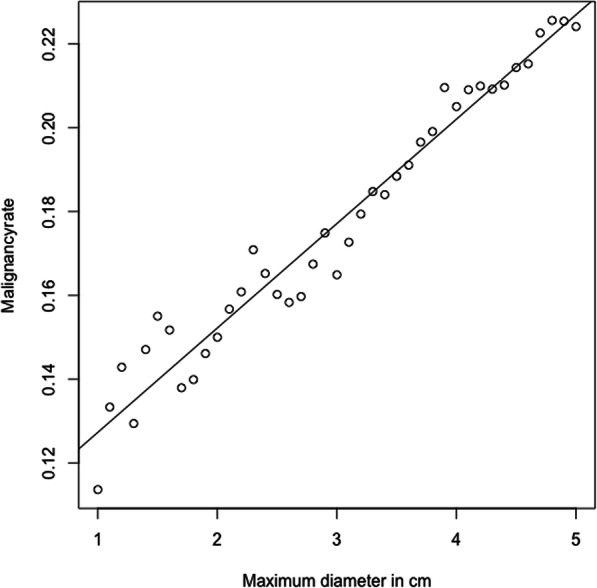
Regression analysis for malignancy rate as function of maximum diameter

Overall, the malignancy rate (malignant vs benign + non-neoplastic masses) was at 22.41% for STMs ≤ 5 cm. The malignancy rate dropped significantly to 16.49% when assessing lesions ≤ 3 cm (*P* = 0.007) and to 15.0% when assessing lesions ≤ 2 cm (*P* = 0.006). The regression analysis for the malignancy rate as a function of maximum diameter is shown in Fig. 1. The malignancy rate of superficial STMs was at 23.53% (STMs ≤ 3 cm: 19.19% / STMs ≤ 2 cm: 18.46%) and of deep STMs at 22.14% (15.32% / 13.79%), with no significant difference between the three groups (*P* = 0.97 / *P* = 0.49 / *P* = 0.95). Nevertheless, the malignancy rate for STMs ≤ 5 cm varied depending on tumor localization as shown in Table 4 (*P* < 0.001). The foot region showed the lowest and the head and neck region the highest malignancy rate. STMs located in the head and neck were the only subgroup of lesions which showed a tendency towards an increasing malignancy rate with decreasing tumor diameter.

**Table 4:** Malignancy rates of soft tissue masses ≤5 cm, ≤3 cm and ≤2 cm in different locations

Malignancy rate	≤ 5 cm	≤ 3 cm	≤2 cm
Overall	22.41%	16.49%	15.0%
Superficial	23.53%	20.79%	18.18%
Deep	22.14%	15.32%	13.79%
Head / Neck	48.57%	71.43%	80.0%
Trunk	40,00%	33.33%	45.45%
Upper Extremity (excluding Hand)	25.27%	24.21%	20.45%
Lower Extremity (excluding Foot)	20.7%	11.22%	11.11%
Hand	19.3%	19.15%	16.67%
Foot	7.44%	5.68%	3.70%

The malignancy rate was significantly lower in age group B as compared to age groups A and C for lesions ≤ 5 cm, ≤ 3 cm, and ≤ 2 cm (*P* < 0.001, *P* = 0.003 and *P* = 0.022). Percentages are shown in Table 5.

**Table 5:** Malignancy rates of soft tissue masses for age groups A (< 18 years), B (18–40 years), and C (> 40 years)

Malignancy rate	≤ 5 cm	≤ 3 cm	≤2 cm
Age group A (< 18 years)	38.89%	19.05%	23.08%
Age group B (18–40 years)	12.46%	8.07%	5.88%
Age group C (> 40 years)	26.73%	20.27%	18.31%

## Discussion

In this study, we evaluated the distribution of entities of soft tissue tumors with a maximum diameter of 5 cm in a large series of a tertiary referral center and give an overview of expected entities and likelihood of malignancy of small lesions in initial diagnosis.

The distribution of entities with lipoma and tenosynovial giant cell tumor as most common non-malignant lesions and undifferentiated pleomorphic sarcoma and liposarcoma as most common malignant lesions in general is in line with previous studies [10, 12, 13].

Before this study, in 1995 Kransdorf et al. were the last to give an overview over the distribution of entities of soft tissue masses in a large scale. While Kransdorf et al. included a high number of lesions in their study, lesion size and rates of malignancy for different sizes were not assessed in this previous publication [12].

In this study malignancy rates were analyzed in 870 lesions ≤ 5 cm. Although the malignancy rate of all lesions ≤ 5 cm was around 20% and dropped significantly when assessing lesions ≤ 3 cm and ≤ 2 cm, still almost every seventh lesion with a diameter of less than 2 cm was malignant. Decrease of malignancy rate with decrease of lesion diameter is in line with studies published by Chen et al. and Obaid et al. including lesions of all sizes evaluated on MR images [6, 11].

When focusing on lesions ≤ 2 cm a malignancy rate of 15.0% was observed, whereas Pham et al. published a study focusing on STMs of this size describing a malignancy rate of 21.42% [10]. The number of lesions assessed in this previous study was however considerably smaller compared to our series (*n* = 42), possibly accounting for the observed variance in malignancy rate. Hsieh et al. also performed an analysis of soft tissue masses, showing significantly lower malignancy rates. In contrast to our study, lesions of all sizes and especially tumors of the skin were included [14]. Overall, the results of our study show that, despite relatively low rates of malignancy, even smaller lesions should be considered as potentially malignant [6, 11].

Of 870 lesions ≤ 5 cm analyzed in our study, the majority was located in the deep soft tissues.

Although the comparison between superficial and deep lesions revealed a different distribution of entities, no significant difference in malignancy rates was observed. These results are in line with those of Datir et al. who also did not observe any influence of lesion depth on malignancy rates comparing STMs of any size on MR imaging [7]. Therefore, the possibility of malignancy of small lesions needs to be considered regardless of the lesion being located superficial or deep to the fascia.

The malignancy rates for patients younger than 18 years and older than 40 years were significantly higher as compared to patients between 18 and 40 years of age. In the group of young patients this was caused by the peak of alveolar soft part sarcoma and synovial sarcoma in adolescence, in patients older than 40 years this was mainly due to the higher percentage of liposarcomas and undifferentiated pleomorphic sarcomas [15].

Furthermore, significant differences of malignancy rates were observed with a view to tumor location in different regions of the body: Lesions of the head and neck showed a relatively high rate of malignancy, whereas soft tissue masses located in the feet showed relatively low malignancy rates. The relatively low malignancy rates in the hand and foot might be caused by the high number of tenosynovial giant cell tumors occurring in these regions [16]. Nevertheless, also in these peripheral locations still a considerable number of malignant lesions was found.

Overall, the results of this study show, that independent of the localization of a soft tissue mass it is important to consider even small lesions as potentially malignant. Therefore, both diagnostics and therapy of unclear soft tissue lesions should generally be performed in specialized tumor centers, since ignoring small soft tissue masses or performing primary resection with failure to achieve tumor-free margins can lead to a poor outcome.

Additionally, for physicians initially confronted with patients with small tissue lesions on clinical examination, ultrasound, CT, or MR imaging, this study gives an overview on the distribution of entities depending on size, localization, and depth, and therefore allows an estimation of what can be expected.

This study has limitations. As our institution is a tertiary referral center, there is a selection bias toward malignant lesions with predominantly unclear or likely malignant lesions being referred to our institution and majority of benign lesions being followed up or treated in peripheral institutions. This bias may be enhanced by the fact, that we only investigated lesions that underwent biopsy/resection and thus, had a final histopathological diagnosis. For this reason, the distribution of entities seen in a different clinical setting may differ from the results of this study [17]. Moreover, results may differ from those of other comparable institutions due to different regional and practice-dependent referral patterns [3, 18]. Finally, no further evaluation of MR characteristics was performed due to the large sample size and the inhomogeneity of MR examinations obtained at different institutions. A narrowing of the differential diagnosis would have likely been possible by analyzing the full MR morphology rather than tumor size and localization only.

## Conclusions

In conclusion, the rate of malignancy decreased significantly with tumor size in both, small superficial and deep STMs. Nevertheless, even small STMs have a considerable likelihood of malignancy and therefore, it is crucial that diagnostics and therapy are performed in specialized institutions. The distribution of entities was different between superficial and deep STMs, yet there was no significant difference found between the two groups regarding the malignancy rate. This study may help to better estimate the distribution of entities and the probability of malignancy of superficial and deep STMs of small size and consequently, to decide whether a biopsy or primary resection should be chosen and to avoid the failure to achieve tumor-free margins.

## Data Availability

All required data is available within the manuscript.

## Abbreviations

AJCC: American Joint Committee on Cancer
MR: Magnetic resonance
STM: Soft tissue mass
WHO: World Health Organisation
